# Responses to provision of personalised cancer risk information: a qualitative interview study with members of the public

**DOI:** 10.1186/s12889-017-4985-1

**Published:** 2017-12-22

**Authors:** Juliet A. Usher-Smith, Barbora Silarova, Artitaya Lophatananon, Robbie Duschinsky, Jackie Campbell, Joanne Warcaba, Kenneth Muir

**Affiliations:** 1 The Primary Care Unit, Department of Public Health and Primary Care, University of Cambridge, Box 113 Cambridge Biomedical Campus, Cambridge, CB2 0SR UK; 2 MRC Epidemiology Unit, Institute of Metabolic Science, University of Cambridge, Cambridge, CB2 0QQ UK; 3 Institute of Population Health, University of Manchester, Oxford Road, Manchester, M13 9PL UK; 4 Institute of Health and Wellbeing, University of Northampton, Park Campus, Boughton Green Road, Northampton, NN2 7AL UK; 5 Moulton Surgery, 120 Northampton Lane North, Moulton, NN3 7QP UK

**Keywords:** Cancer, Risk, Communication, Prevention, Qualitative research

## Abstract

**Background:**

It is estimated that nearly 600,000 cancer cases in the UK could have been avoided in the past five years if people had healthier lifestyles. A number of theories of behaviour change suggest that before people will change health behaviours, they must accept that a risk applies to them. This study aimed to explore the views of the public on receiving personalised cancer risk information and the potential for that information to motivate behaviour change.

**Methods:**

We conducted 27 interviews with members of the public (mean age 49 ± 23 years). Each participant completed a questionnaire to allow calculation of their risk of developing the most common cancers (10 for women, 8 for men). During the interviews we presented their risk using a web-based tool developed for the study and discussions covered their views on receiving that information. Each interview was audio-recorded and then analysed using thematic analysis.

**Results:**

Participants generally viewed the concept of personalised cancer risk positively. The first reaction of almost all when presented with their 10-year risk of an individual cancer without any further context was that it was low and not concerning. Views on what constituted a high risk ranged widely, from 0.5 to 60%. All felt seeing the impact of changes in lifestyle was helpful. For some this led to intentions to change behaviour, but reductions in risk were not always motivating as the risks were considered low and differences small.

**Conclusions:**

Provision of personalised cancer risk was well received and may be a useful addition to other cancer prevention initiatives. Further work is needed in particular to develop ways to present cancer risk that reflect the general perception of what constitutes a risk high enough to motivate behaviour change and help patients contextualise a less well known health risk by providing a frame of reference.

**Electronic supplementary material:**

The online version of this article (10.1186/s12889-017-4985-1) contains supplementary material, which is available to authorized users.

## Background

Up to 40% of all cases of cancer are potentially preventable through changes to lifestyle factors such as smoking, alcohol consumption, diet, weight and physical activity [1]. We know from evidence, however, that only 3% of the general public are aware that being overweight can increase their risk of cancer, less than a third are aware that physical activity could help reduce risk [2–5], and one in seven people believe that lifetime risk of cancer is unmodifiable [6]. Additionally, when asked to estimate their risk of cancer, many individuals have incorrect risk perceptions [7–9].

A number of behaviour change theories, including Protection Motivation Theory [10] and the Extended Parallel Process Model [11], suggest that providing individuals with their estimated risk of developing cancer and demonstrating the impact of lifestyle change on that future risk may motivate change at an individual level through increases in accuracy of risk perception and response efficacy [12]. This could then complement wider collective approaches to shifting population distributions of behaviour and risk factors. A number of risk tools are now available which predict an individual’s future risk of cancer and which could be used for this purpose [13–21]. Previous research has confirmed that providing cancer risk information to individuals can improve accuracy of risk perception [9, 22, 23] and enhance response efficacy [24] and intention to have cancer screening [25, 26]. However, most studies exploring preferences for formats of presentation and responses to cancer risk information have used hypothetical cases [27–30] and there remains uncertainty over the views of the general public on being offered their own cancer risk and how best to present risk to promote understanding and motivate behaviour change. Unlike similar risk tools for other conditions, such as QRisk2® [31] for cardiovascular disease, these tools are also rarely used in routine healthcare settings [32].It is therefore not clear how best to incorporate risk assessment into practice to maximise benefits whilst minimising harms.

The aim of this study was to address some of these uncertainties by exploring the reactions of members of the general public to different presentations of estimates of their own personalised cancer risk and their views on whether such information should be incorporated into routine medical practice.

## Methods

Face-to-face interviews were conducted with members of the public in Cambridge, UK. Ethical approval was obtained from the East of England Cambridgeshire and Hertfordshire Research Ethics Committee (reference 15/EE/0310). All participants provided written consent.

### Participants and recruitment

To enable us to include individuals with a range of backgrounds and of different ages we used three recruitment strategies: 1) posters in the waiting rooms of ten Primary Care Practices across Cambridgeshire; 2) letters of invitation and the participant information leaflet sent via the lead of the group to a local patient participation group; and 3) letters of invitation and the participant information leaflet sent by Practice staff to 100 randomly selected patients aged between 18 and 65 years and registered at one Primary Care Practice in Cambridgeshire. By sending letters of invitation to a local patient participation group and to individual patients at one Primary Care practice, we were able to reach potential participants who may not have accessed healthcare during the recruitment period. For those responding to the posters or invitation letters via the patient participation group, no age limits were applied. The letters of invitation to individual patients were sent out part way through recruitment and the decision to limit to an upper age of 65 years was made to purposively sample younger people. In all cases those interested in taking part in the study were invited to contact the research team and all those who completed the pre-interview questionnaire and agreed to an interview were included in the study. Letters of invitation to patients registered at the Primary Care Practice were sent out in batches of 20 and we continued recruitment until we reached data saturation.

### Pre-interview questionnaire

Prior to the interviews, participants provided their address and were posted a screening questionnaire asking about their risk factors for cancer. This included questions about age, sex, family history of cancer, medical history and lifestyle factors such as smoking, alcohol consumption, diet, weight and physical activity. Responses were used to i) calculate an individual’s risk of developing the cancers specified below; and ii) contextualise findings from the subsequent interviews. All those with a previous diagnosis of cancer were advised at this stage that the risk algorithms being used had not been tested in those with a previous diagnosis of cancer and so might either underestimate or overestimate their risk. Socioeconomic status was computed using the participants’ postcode and the English indices of deprivation 2010 available online [33].

### Calculation of risk

To calculate the absolute and relative risk of individual cancers for each participant we adapted the risk algorithms used in the “YourDiseaseRisk” models [13] for 11 cancers (female breast, colorectal, stomach, bladder, prostate, kidney, melanoma, ovarian, lung, pancreatic, and uterine) for the UK population. These models were chosen as they have a consistent approach across multiple cancer sites. Full details of the method used to adapt the algorithms are published elsewhere [34]. In brief, we first estimated the prevalence in the UK population for each of the risk factors related to each cancer type and used that data alongside the “YourDiseaseRisk” algorithms to calculate an individual’s risk of developing each of the cancers relative to the average UK population.

To enable calculation of the 10-year estimated absolute risk for all 11 cancers we used the “Current Probability” method [35] based on data from the Cancer Research UK website [36] for age- and sex-specific cancer incidence and data from the Office of National Statistics [37] for age- and sex-specific death rates from all causes. This allowed us to estimate the average age- and sex-specific UK 10-year absolute risk for each cancer and we multiplied this by each individual’s relative risk to obtain the estimated 10-year risk for individuals.

### Presentation of risk

To enable communication of the risk to participants we developed a web-based tool that presented the estimated risk for each cancer either individually or alongside others. In order to choose the formats in which to present the risk we first reviewed published literature [27, 38–40] and the internet to compile an inventory of different formats currently in use for cardiovascular disease and cancer. Informed by best practice guidance for communication of risk [41, 42] we then met with patient and public representatives to discuss a range of formats and choose those to use for the study.

**Fig. 1:**
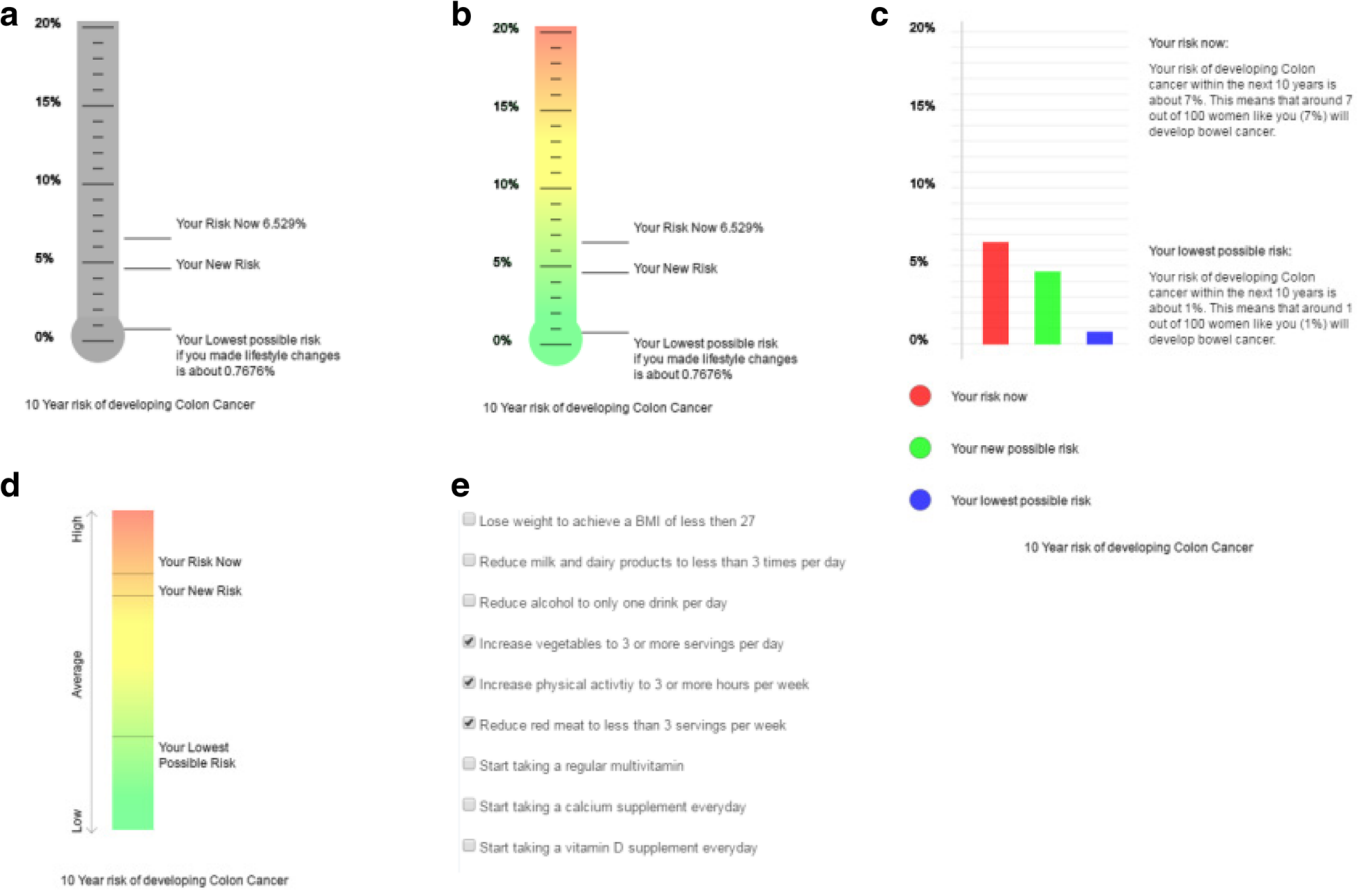
Formats of risk presention. **a**) absolute 10-year percentage risk on a vertical thermometer scale in grey-scale; **b**) absolute 10-year percentage risk on a vertical thermometer scale coloured from green to red; **c**) absolute 10-year percentage risk as a coloured bar chart; **d**) relative 10-year risk on a qualitative scale from low to high; **e**) options to see the impact of changes to lifestyle on risk provided alongside each risk format

The chosen formats are shown in Fig. 1. They are: a) absolute 10-year percentage risk on a vertical thermometer scale in grey-scale; b) absolute 10-year percentage risk on a vertical thermometer scale coloured from green to red; c) absolute 10-year percentage risk as a coloured bar chart shaded; and d) relative 10-year risk on a qualitative scale from ‘Low risk’ to ‘High risk’. All participants were shown the formats in the same order for either breast or colorectal cancer to allow us to assess the impact of colour over a grey-scale image. In all formats participants were given the opportunity to select changes to their lifestyle such as those shown in panel e of Fig. 1 and see the impact of those changes on the calculated risk of that cancer. They were then given the option to view up to six cancers of their choice from those available simultaneously. Participants with a previous diagnosis of cancer were not given the option to see their risk of that specific cancer.

### Data collection

The one-to-one face-to-face interviews were conducted by one of two female researchers (JUS, an academic GP or BS, a psychologist; both with PhDs and experience of conducting qualitative research) and, depending on the participant’s preference, held either in the participant’s home (*n* = 11) or in a room at the University of Cambridge (*n* = 16). The interviews were semi-structured and based on a schedule covering their views on receiving personalised risk information in general, of different representations of that risk, the potential for motivating lifestyle change, and the timing and site of delivery of the information (see Additional file 1). Each lasted between 20 and 50 min and at the end of each interview the participants were offered a summary of their risk of the individual cancers and Cancer Research UK leaflets on how to reduce their risk and how to recognise signs and symptoms of cancer. The background of the researchers was not discussed during the interviews.

### Analysis

The interviews were audio-recorded and transcribed verbatim and analysed using thematic analysis [43] and the one sheet of paper (OSOP) method [44]. After approximately half of the interviews had been completed, the transcripts were first read and re-read by three researchers (a UK academic GP, a health psychologist and a social scientist). A coding frame (available from the authors) based on the transcripts and the interview schedule and informed by relevant concerns in the literature was developed by those same researchers. One researcher (a UK academic GP) then coded all the transcripts with the aid of NVivo software (QSR International, version 10). Once coding was complete, we created a written summary of the data for each code using the OSOP (one sheet of paper) method where every section of data relevant to that code from all the interviews was noted. These were discussed amongst the wider research team to identify the key themes first within each code and then across the entire dataset and any outlying views. These summaries were added to as further data were collected and once all data had been collected and summarised we then explored these themes in depth, looking for any patterns across different ages, sexes, current lifestyle habits, and personal and family history. Throughout the text illustrative quotes are identified by sex and age group.

## Results

We conducted interviews with 27 participants. The demographic and selected lifestyle characteristics of the participants are shown in Table 1. 11 were male and 16 female and the mean age was 49 ± 23 years. Most were white British from areas of low deprivation with no personal or family history of cancer and high levels of education. The majority were already physically active though half had a BMI of over 25 kg/m^2^ and half did not meet the recommendation of eating five or more servings of fruit or vegetables per day [45]. When asked about their views on cancer risk prior to the study, approximately half reported they were concerned about their own risk of cancer whilst the other half were either not concerned or had not really thought about it.

**Table 1:** Characteristics of participants who took part in interviews exploring their views of the provision of personalised cancer risk information

	*n* = 27
Age (years)	
20–30	4
30–40	10
40–60	5
> 60	8
Mean ± SD	49 ± 23
Sex	
Male	11
Female	16
Ethnicity	
White British	21
Asian	5
Other	1
Index of multiple deprivation tertile	
1 (least deprived)	16
2	9
3 (most deprived)	2
Highest education level	
University degree	22
A levels	1
College	3
Missing	1
Personal history of cancer	
Yes	3
No	24
Family history of cancer in a first degree relative	
Yes	8
No	19
BMI (kg/m^2^)	
< 20	3
20–25	11
> 25	13
Smoking status	
Non-smoker	20
Ex-smoker	7
5 or more servings of fruit or vegetables per day	
Yes	14
No	13
Moderate physical activity for at least 30 min on most days or 3 h per week	
Yes	22
No	5

### Initial reaction

The first reaction of almost all participants to being presented with their 10-year absolute risk of either breast or colon cancer in grey-scale was that it was low and they were either reassured or happy with the results and not concerned or worried.

*“Well, it’s not even 1%. I just think that’s really good, out of 100%, I don’t really need to worry about colon cancer, that’s what I would say.”* (F,30–40).

In some cases these impressions were clearly related just to the fact that the number was small or to the position on the scale rather than an appreciation of the meaning of the number itself.

*“Good, I’m relieved. Just seems a low number. I don’t know whether it is or not, but, is it a low number?”* (M,>60).

*“Okay, that’s quite low then... ‘cos it’s at the very bottom of the scale. Yeah, it’s at the bottom so quite happy with that.”* (F,20–30).

Only one participant thought the risk was fairly high and higher than they had expected.

### Perception of what would be high risk

When asked what would constitute a high risk for them there was a wide range of responses from 0.5% to 60%. The most common were 10% (*n* = 7), 20% (*n* = 4), 5% (*n* = 3), and 1% (*n* = 3) which together accounted for over half of the responses. There was no association with age or deprivation.

Particularly noticeable was the finding that over half were unclear about why they had chosen the number they had. Some individuals were explicit in saying that they just liked the sound of the numbers they chose whilst others found it difficult to explain their reasoning.

*“I don’t know, it’s just something that, yeah, the number that just came into my head”* (F,30–40).

A small number of participants related it to something in daily life or other diseases. In general, those who related it to something specific tended to choose lower, more accurate, numbers than those who didn’t.

*“1% is quite a high risk for many things that, if you don't have to take it you wouldn't take it. I mean, would you choose to cross the road if one time in a hundred it happened? No, you'd find a different way*.” (M,30–40).

*“I would have probably compared it to cardiovascular disease risk which would be, currently it’s 10% over 5 years isn’t it I think so I guess I would have viewed it as the same figure for cancer.”* (M,>60, history of cancer).

### Views on format of risk presentation

#### Preference for colour

Most participants had a preferred format of risk presentation but approximately equal numbers preferred the coloured thermometer, the bar chart and the relative risk scale. There was, however, a clear preference for colour, and for many the addition of colour dominated interpretation of the number.

*“If it’s on the green I’m like “Phew, that’s good that”…I didn’t even need to look at that percentage.”* (F,40–60).

Notably two participants did not like colour. One felt it was ‘childish’ and potentially gave conflicting information if the number was low but coloured red and the other felt that by adding colour we were deliberately trying to manipulate their interpretation and that made them ‘sceptical’ about the risk.

#### Benefits of being able to demonstrate change

All participants were enthusiastic about being able to interact with the risk scores and visually see the potential impact of changes in lifestyle.

*“If you can physically see that that moving down and knowing that, okay, at the moment my risk is say half a percent, if I reduce the amount of red meat that I eat I’ll go down to 0.25% then that’s a good motivational thing to make me do that.”* (F,30–40).

In particular it was felt to be helpful to enable them to choose which aspect of their lifestyle they should focus on first.

*“If I was to lose weight it’s making a difference of about 0.5, and what would be interesting if I then combine that with less meat I get a bigger saving obviously, if I do less meat on its own I get about the same. What I’m trying to see is, you know, there’s five factors and each one of them is an improvement but I don’t want to become a monk and I could probably do two of them, which would be best two to do?”* (M,40–60).

A few participants suggested that it would also be helpful to show the effect of unhealthy choices so they could see what effect their current healthy lifestyle was having and what would happen if they stopped exercising or put on weight, for example. Another participant felt having smaller increments of change might help those needing to make large changes. For example including an hour of physical activity per week rather than just the recommended three hours.

#### Absolute versus relative risk

Almost all participants responded positively to seeing their risk relative to people of their age and sex in the UK population as it enabled them to put their risk ‘into perspective’. Two participants, however, were not interested in comparing themselves to others as they considered their risk personal to them.

*“I don’t know that I would prefer it, I would say it’s useful information, but I think to me personally I am not really fussed where I am compared to average…”* (F,30–40).

#### Individual versus multiple cancers

All participants liked being able to see their risk of multiple individual cancers alongside each other and spent time during the interviews selecting and deselecting cancers and lifestyle options to compare their risk of different cancers and the effects of lifestyle changes. Notably many had specific cancers that they were most interested in, often those which had affected family members or friends. When asked whether they preferred to see them individually or combined, a small number felt strongly that they would prefer individual cancers because ‘*different cancers are caused by different things*’ (F,>60) or just one combined risk because they ‘*didn’t distinguish different types of cancer*’ (M,30–40) and felt all cancers were bad so were primarily interested in reducing their overall risk. The majority expressed preference for both an overall combined risk and the option to look at individual cancers.

*“I think it would be nice to have combined risk but if you break it down to individual you can then see which cancer has got the highest risk and then you can then from that choose to change your lifestyle to reduce that, so you can reduce that particular one.”* (M,>60).

### Potential for personalised cancer risk information to motivate behaviour change

Most participants felt personalised cancer risk information would help others to make changes to their lifestyle.

*“I definitely think it's helpful and I think it's gonna really motivate people to make changes because, yeah, cancer's scary and if you see that your risk can drop it, yeah, I think it might really motivate people to make changes.”* (F,30–40).

A number also reported intentions to change their own behaviour after receiving their personalised cancer risk. For some this was because seeing their risk reaffirmed plans for changes they had already been considering. For others the motivation came directly from now wanting to lower their risk.

*“... for me it reaffirms that my choices and changes that I’d like my lifestyle and it’s actually in the right direction and it will certainly help me to stick to them and continue with these choices for the perceived future.”* (M,30–40).

*“It motivates me now to decrease my risk.….I will try to start lifestyle changes now, yes….I’d like to lose some weight, and eat, you know, healthily.”* (F,30–40).

There were also several who described a balance with some things being easier to achieve and so possible at a lower risk and some that would require a higher risk in order to motivate them.

*“Reduce red meat, I guess I could try, maybe I would, so I would think, okay, so that’s my risk but it says less than three servings per week, I could do that I think or at least get a, go towards that, you know…*.. *Eat the vegetables no, I’m afraid not*.” (F,40–60).

However, for many, receiving their personalised cancer risk did not influence their intention to change their behaviour. Reasons for this fell into three groups (Table 2). The first, and most common, was that the risk was perceived to be so low that reducing it further was not a priority. The second, who tended to be those with the lowest risk, were reassured that they were already living a healthy lifestyle and so did not feel they needed to make any changes. The third group were older participants who felt that it was too late to start worrying about cancer, either because they felt it was simply too late to change or because they accepted that they had to die from something.

**Table 2:** Reasons for not expressing any intention to change behaviour

Reasons for not expressing any intention to change behaviour	
Risk so low	*“So that’s interesting, you’re right, I could get it down to absolutely zero if I lived as a hermit, and I think that’s the point..… if my risk factor was very high, very high, I would probably do something about it, but because it’s so low, then actually getting it even lower with having to do significant things to my lifestyle, then I probably wouldn’t. There, that’s my balance.”* (M,40–60) *“I mean all of the values are in some ways very theoretical because it’s all low risk and I suppose I’m not doing anything that is considered high risk, so I would probably not, so not be worried by the things I’ve seen and sort of spurned on to make lots of changes because it’s all low risk.”* (F, 30–40)
Reassured that already living a healthy lifestyle	*“I’m pretty happy with my low risk of cancer and I’ll just keep doing as I have been doing with my lifestyle behaviours.”* (F,20–30) *“I mean very informative and I think everybody should know these risks, but for me it’s not rung alarm bells, I just keep on, keep on eating healthy, keep on exercising regularly and having a good balanced lifestyle,* etc.*,* etc.*,”.* (F,40–60)
Not worried about risk – too late at my age	*“I think it’s too late probably, when I was 50 it would be different but now I think it’s too late to change much”.* (F,>60) *“I wouldn’t think of doing anything about it because at my stage of life, let’s suppose there was alright a 10% chance that within the next 10 years I’ll get cancer and we think, right, I can avoid that by doing A, B and C but thank you, what are the other options because I’m going to get one of them, so that’s why it doesn’t worry me on the basis that I’m assuming there is at least a 1 in 10 chance if not higher I will die within the next 10 years, so you start looking at what am I going to die of and my immediate reaction is I don’t see I can be confident that the alternatives will be less unpleasant.”* (M,>60)

### Views on provision of cancer risk information generally

All participants were supportive of offering cancer risk information more widely and would recommend it to their family and friends. There was a clear distinction between offering and providing though, with recognition that it should be up to the individual and may not be appropriate for everyone, particularly those who are already very health-conscious.

When asked how they would prefer to receive this information outside the context of a research study the majority expressed a preference for face-to-face with the others preferring it online or online with the option to discuss it face-to-face. Reasons participants gave for preferring face-to-face delivery were that it provided an opportunity to ask questions and confirm their interpretation and that looking at it at home alone for the first time had the potential to be scary and cause panic. Some also felt receiving the risk in person was more likely to motivate people to make changes to their lifestyle.

*“I think face-to-face is better you know in a general sense for any communication, just from my experience I think whatever you do, face-to-face is much more, it’s more likely to make it have an effect I think.”* (M,>60, history of cancer).

Many mentioned their Primary Care physician (PCP) or other primary care healthcare professionals as the most appropriate people to deliver the information. However, several were concerned that PCPs would not have the time or should focus on other aspects of care. There was a general view though that, if the information was not being given face to face by the their PCP or another health care professional, it needed to be endorsed by the PCP to provide credibility.

## Discussion

Most previous studies to date in this area have focused on preferences for different formats of presentation using hypothetical examples [27–30] or on measuring accuracy of perceived risk after provision of risk information [9, 46–48]. By providing individuals with their own personalised risk estimate within the context of a research interview, this study provides novel insights into the reactions of the general public to receiving their personalised estimated risk of cancer. We found that participants generally viewed receiving this information positively and were keen that it be provided more widely. When presented with their 10-year absolute risk of an individual cancer on a grey-scale almost all felt that it was low and not concerning. Views on what constituted a high risk ranged widely, from 0.5 to 60%. When presented in colour, the colour was often more important than the number and dominated their interpretation but there was no clear preference for one format of presentation. All felt seeing the impact of changes in lifestyle on their risk was helpful. For some this led to intentions to change behaviour, but reductions in risk were not always motivating as the risks were considered low and differences small.

Perhaps the most important of these findings is that whilst many of the participants described how their intention to change their behaviour was influenced by their risk, a finding consistent with many behavioural theories, they appeared to be influenced less by a specific image of their cumulative chance of cancer and more by a sense of what is a “significant” number in general. As a result, many had very high thresholds compared to the estimated risks of individual cancers. Improving accuracy of risk perception alone may, therefore, not influence behaviour and the need for either relative risk information or colour grading to provide context for interpretation of risk [27, 28] is particularly important for cancer when risks are generally low.

The finding that there was no clear preference for the format of risk presentation is consistent with other studies in which participants were provided risk of cancer or cardiovascular disease in different formats [27–29] and highlights the need for a range of individualised presentations that take into account the numeracy and graph literacy of the audience [42]. As in those studies, there was, nevertheless, a clear preference for colour with green seen as safe regardless of the number [28].

Being able to see the impact of changes in lifestyle was felt to be useful by participants, especially in being able to prioritise particular forms of behaviour change. For some, this either supported existing lifestyle choices or encouraged them to introduce new changes. This is consistent with existing research which has demonstrated an association between response efficacy, the belief that an intervention or action is effective against a perceived health threat, and intention to change behaviour [49–52]. The provision of cancer risk information may, therefore, be a useful tool to promote intentions for behaviour change for cancer prevention in some individuals but this requires further confirmation from studies with quantitative study designs.

### Limitations

These findings must, additionally, be interpreted with consideration of the limitations of the study. The main limitation is that, by definition, the participants were a small, self-selected group interested in their cancer risk as they had responded to invitations to take part in a research study to help the development of personalised cancer risk tools. We attempted to reduce this bias by using three different recruitment strategies and approximately half of the final sample reported either not being concerned about their cancer risk prior to the study or having not really thought about it. Nevertheless we cannot exclude the possibility that the self-selected nature of the sample impacted on the results. The sample also included participants with a wide range of ages and three participants with a history of cancer. This was a deliberate decision at the time of recruitment in order to capture the views of all those to whom cancer risk may be provided in the future, including those with a diagnosis of cancer where there is increasing evidence that lifestyle change can improve survival [53–55] as well as prevent a second cancer. Whilst we did not see a clear difference in the views across the different groups, it remains possible that the views of younger participants may change as they get older and those with a personal history of cancer may interpret risk quite differently depending on the cancer they survived. Those with a previous history of cancer were also told that the risk scores had not been tested in people with experience of cancer and so the meaning of the estimated risks to them may have been different from those without a history of cancer. The effect of the presentation of risk on intention to change behaviour may also have been limited by the characteristics of the participants. Most were from areas of low deprivation, were highly educated and were already achieving the physical activity recommendations. Instead of focusing on their own lifestyle, many instead also discussed how useful they thought it would be for other people. This response may have been supported by knowing they were taking part in a study to discuss different ways of presenting cancer risk.

## Conclusions

Overall, our findings show that, amongst the participants of this study, provision of personalised cancer risk information was well received and may be a useful adjunct to other cancer prevention initiatives. The wide range of views on what constitutes a high risk highlights the need for further work to address the disparity between typical risk estimates of individual cancers and the general perception of what constitutes a risk high enough to motivate behaviour change. In addition, this study suggests that when risks of cancer are presented without context many people are likely to consider them low and not concerning. The use of colour can help but the choice of colours need careful consideration. Alternatively, providing patients with the risks of other diseases or life events may help patients contextualise a less well known health risk by providing a frame of reference.

## Additional file

Additional file 1: Interview schedule. (PDF 280 kb)

## Abbreviations

BMI: Body mass index
GP: General practitioner
OSOP: One sheet of paper method
PCP: Primary care physician
