# Supplementary material for: Responses to provision of personalised cancer risk information: a qualitative interview study with members of the public
Source: BMC Public Health. 2017 Dec 22;17:977. doi: 10.1186/s12889-017-4985-1 (PMC5741964; doi:10.1186/s12889-017-4985-1)
Supplement: Additional file 1: — Interview schedule. (PDF 280 kb) [file 12889_2017_4985_MOESM1_ESM.pdf]

**PERSONALISED CANCER RISK STUDY**
**Interview schedule**

**Introduction**

The purpose of this part of the study is to find out about your views on receiving information about risk of cancer. Everything that you say here will be kept confidential, and neither your name nor any other identifying information will be used in any report coming from this research.

1. May I ask you first, at the time when you were invited to take part in this study, what did you know or think about your risk of developing cancer? [Prompts: What was this perception based on? Is it for cancer in general or specific cancers? Have you discussed your risk with a GP or another healthcare professional before?]

2. To what extent have you been concerned about your risk of developing cancer, is this something you thought a lot about? [if yes – were there particular moments or events/incidents that made you think about it?]

3. Are you aware of things that you could do or are already doing to reduce your risk of future cancer? [Prompts: If yes, what and what have they been doing]

**Providing information**

When you agreed to take part in this study you kindly answered a series of questions about yourself, your family and your lifestyle.

4. How did you feel *whilst* answering those questions? [Prompts: easy/difficult, any too personal, how long did it take?]

5. How did you feel *after* answering those questions? [Prompts: anxious, worried, prepared to change lifestyle?]

**Giving risk information**

We have used that information to calculate your risk of developing the 12 most common cancers. First of all I would like to just show you your risk for one of those cancers, bowel cancer, and ask about how you feel receiving the score and what it means to you. I am then going to show you that same risk for bowel cancer presented in different ways to see which you prefer and give you the opportunity to see the effect of changing your lifestyle on the risk and then show you your risk for multiple cancers. At the end of the interview, if you want them, I will give you a copy of your risk for all the cancers and some information about how to reduce your risk and how to recognize signs or symptoms of cancer so you don't need to worry about remembering everything as we go along.

*[Show first presentation of colorectal cancer]*

6. What is your first reaction when you see that? [Prompts: surprised, worried/ relieved]

7. How does it compare to what you thought about your risk beforehand?

8. Different people understand different things when they see information of this kind - what do you understand from it? [Prompts: What do the results mean? Do you think this is a high score, a low score, not high and not low?]

**Different formats of presentation**
I am now going to show you that same information presented in different ways. As you see each one please talk aloud about the thoughts going through your head.

*[Show different formats of presentation of risk]*

If necessary, for each format prompt for:

9. What is your first reaction when you see that?
10. How does it compare to the first/other format(s)?
11. What do you understand by it?

12. Were any of these formats of presentation clearer than the others? Did you find any of them particularly useful? Is there anything that you think may improve the understanding of the risk scores?

13. Which is your preferred presentation? [Prompts: why?]

14. All those formats showed your lifetime risk of developing cancer. Would you have preferred a shorter time period, for example 10 year risk or 5 year risk?

**Seeing the effect of changes on risk**
*[Show preferred presentation of risk]*

By selecting these options it is possible to see how changes in your lifestyle would affect your risk. For example, if you ate more fruit and vegetables/increased the amount of physical activity you do (select as applicable depending on their current lifestyle), your risk would fall to xx. Please go ahead and see the effect of different changes.

15. How do you feel now?

16. Were you aware that changing those factors would reduce your risk of developing bowel cancer? Was anything a surprise?

**Number of cancers**
So far we have focused on one cancer, bowel cancer, but we are able to calculate your risk of 10 different cancers (female) or 8 different cancers (male). This page allows you to choose to see any one of those cancers individually or several at once.

*[Show page with option for up to 10 cancers]*

We have pre-selected the 3 cancers for which you are at highest risk but you can unselect

those or select others.  Please go ahead and choose which cancers you would like to see your risk for.  As you do that, please talk aloud about why you are choosing them.

If necessary, prompt for each:

17. Why did you chose that cancer?
18. Why have you not chosen xx and xx cancers?
19. Why did you unselect that cancer?

*[Show bar chart option of multiple cancers]*

We can also present the multiple cancers like this *[Show multiple cancers from body]*

20. Which of those formats do you prefer?

As with the example earlier with bowel cancer, by selecting these options it is possible to see how changes in your lifestyle would affect your risk.  Please go ahead and see the effect of different changes on the different cancers.

21. How do you feel now?

22. Were you aware that changing those factors would reduce your risk of developing different cancers? Was anything a surprise?

23. Do you think it was helpful to see them all on one page at the same time or would you prefer to look at each one individually or a combined risk for several cancers?

**Views on receiving information in general**

24. Now that you have seen all that information, how do you feel? Do you think it has made any difference to you? Do you think you will change anything in your lifestyle as a result of seeing your risk?

25. Do you think you will discuss your risk with anyone else? [Prompts: family members, GP?]

26. Do you think this information should be available to everyone?

27. If it was not part of a study, how would you have preferred to receive such information? Would you have preferred it in person (e.g. from your GP/nurse)? Or as written information or on the internet? Or alongside other information, for example as part of an NHS Health Check? [Prompts: Why?]

28. Would you recommend to your friends and family to find out about their risk of cancer?

29. Have you been told your risk of any other conditions? [Prompt: Cardiovascular disease, fracture, diabetes]

30. How do you think being given information about future risk of cancer compares to being given information for non-cancer conditions such as heart attack and stroke or diabetes?

**Any other questions and thank you**

31. I have no more questions for you.  Do you have anything else you would like to add?

32. Do you have any questions for me?

33. Thank you very much for taking part
